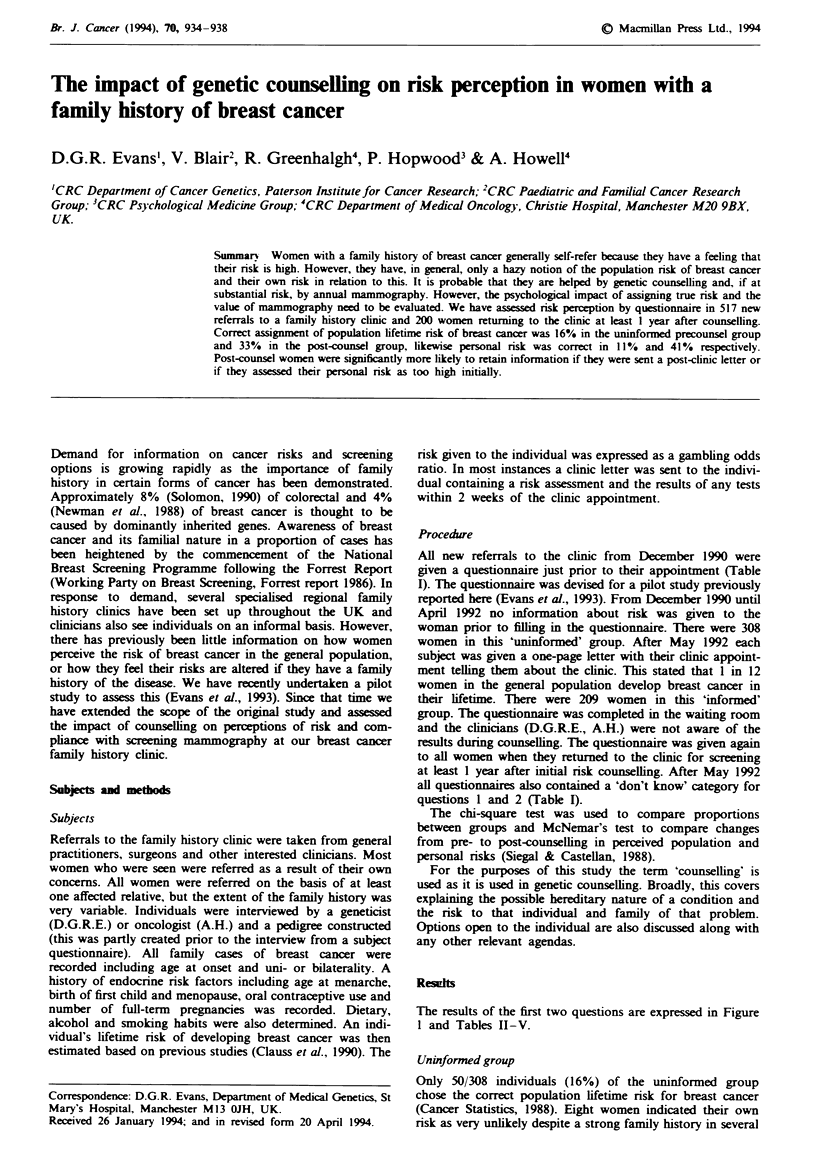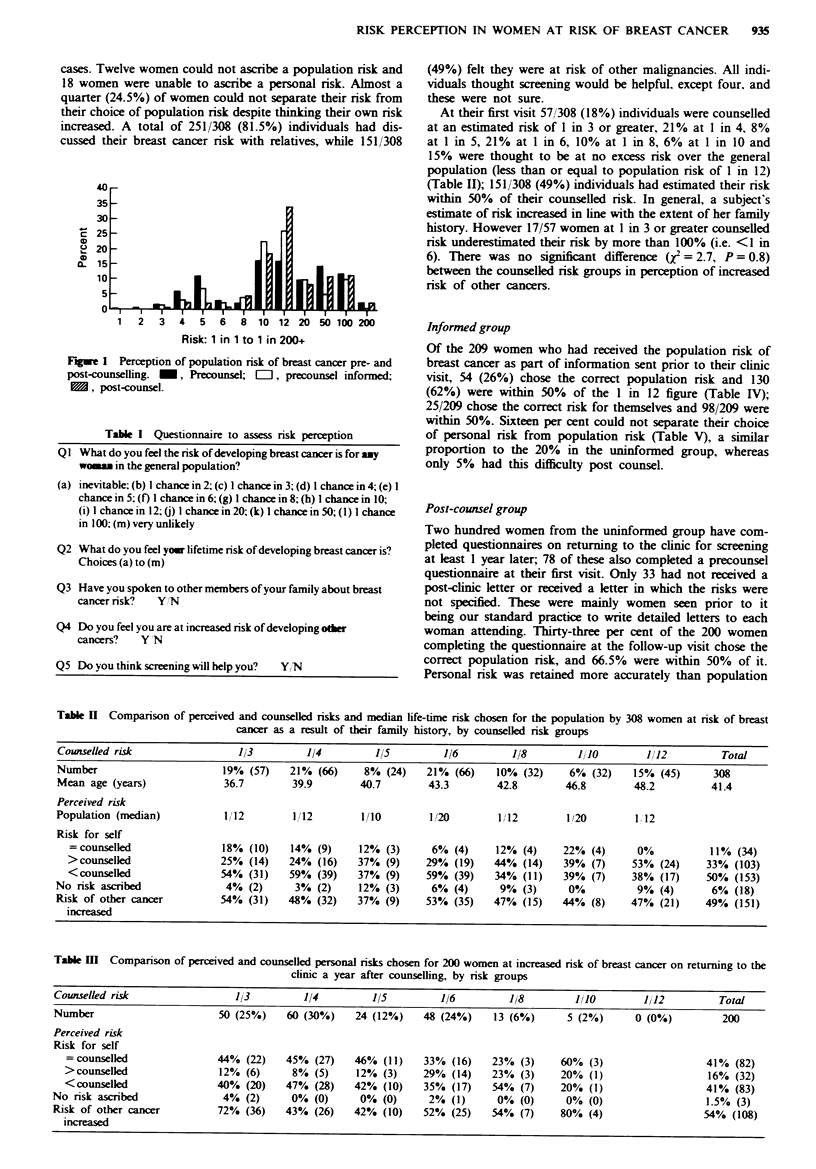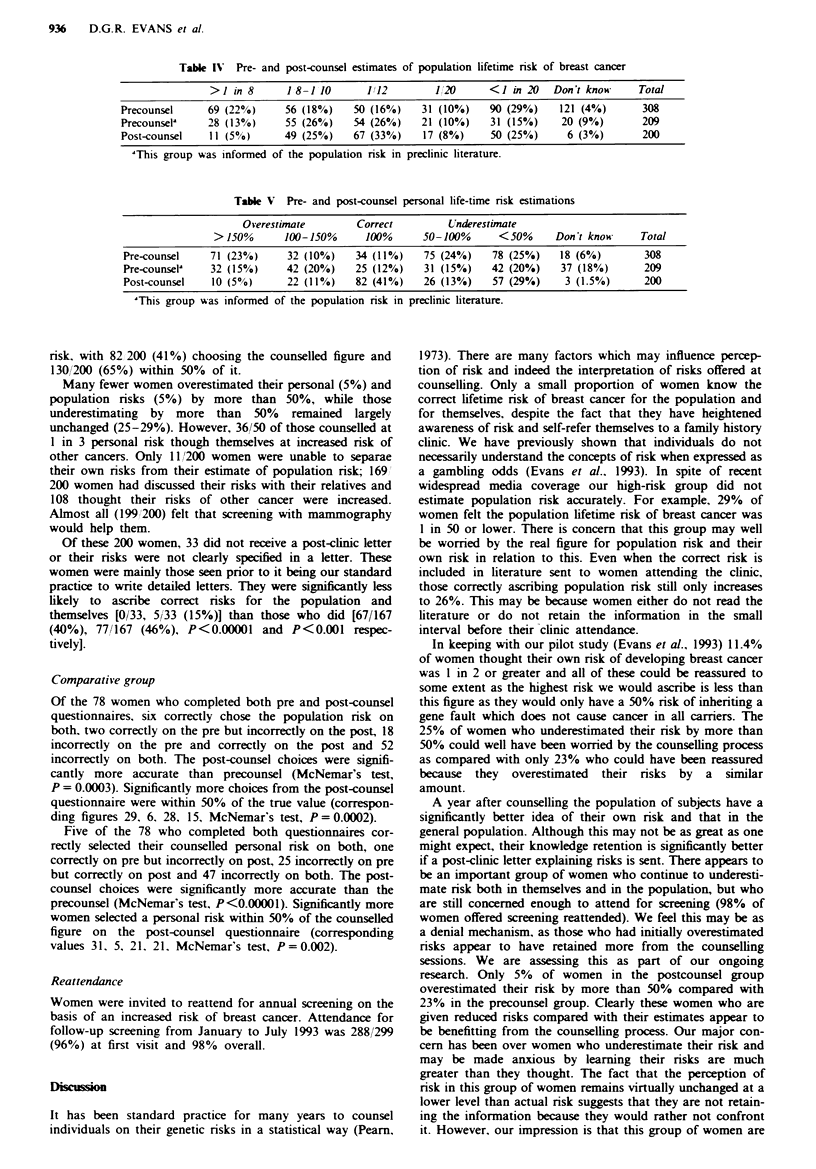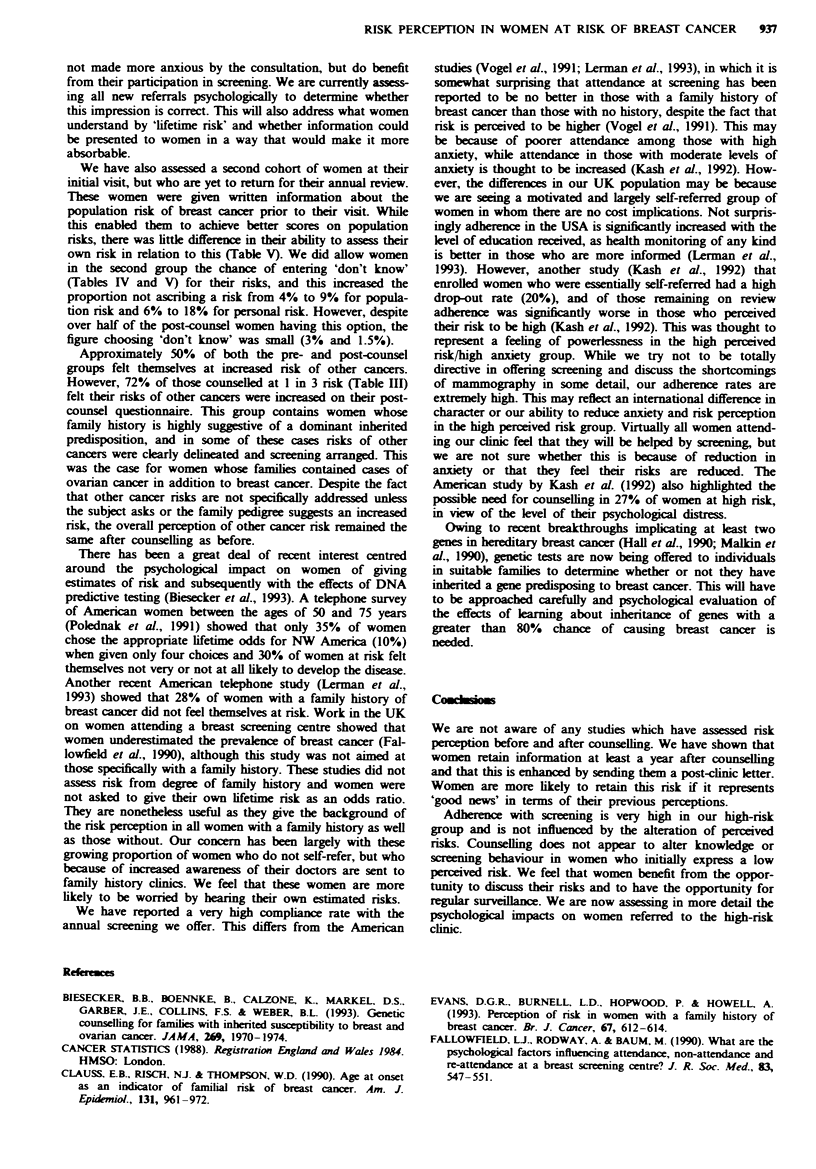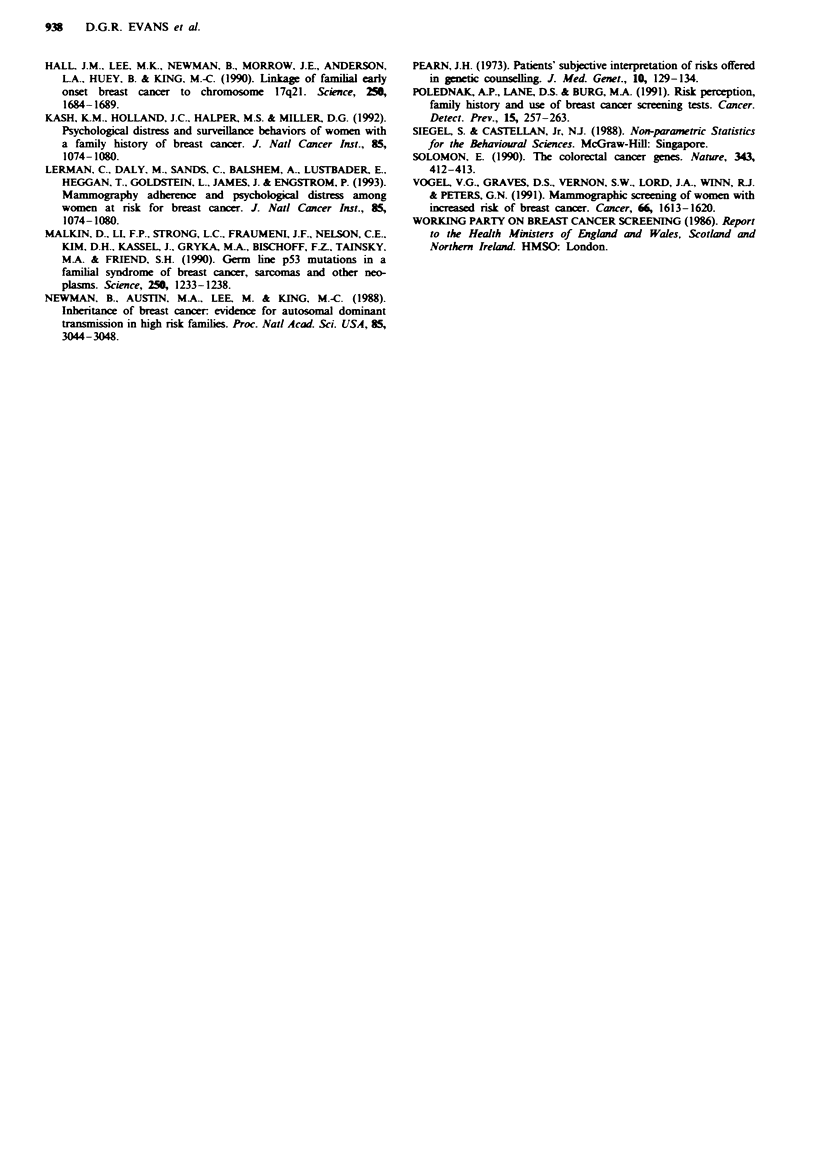# The impact of genetic counselling on risk perception in women with a family history of breast cancer.

**DOI:** 10.1038/bjc.1994.423

**Published:** 1994-11

**Authors:** D. G. Evans, V. Blair, R. Greenhalgh, P. Hopwood, A. Howell

**Affiliations:** CRC Department of Cancer Genetics, Paterson Institute for Cancer Research, Manchester, UK.

## Abstract

Women with a family history of breast cancer generally self-refer because they have a feeling that their risk is high. However, they have, in general, only a hazy notion of the population risk of breast cancer and their own risk in relation to this. It is probable that they are helped by genetic counselling and, if at substantial risk, by annual mammography. However, the psychological impact of assigning true risk and the value of mammography need to be evaluated. We have assessed risk perception by questionnaire in 517 new referrals to a family history clinic and 200 women returning to the clinic at least 1 year after counselling. Correct assignment of population lifetime risk of breast cancer was 16% in the uninformed precounsel group and 33% in the post-counsel group, likewise personal risk was correct in 11% and 41% respectively. Post-counsel women were significantly more likely to retain information if they were sent a post-clinic letter or if they assessed their personal risk as too high initially.


Br. J. Cancer (1994), 70, 934–938 © Macmillan Press Ltd., 1994

### The impact of genetic counselling on risk perception in women with a family history of breast cancer

D.G.R. Evans[1], V. Blair[2], R. Greenhalgh[4], P. Hopwood[3] & A. Howell[4]

[1]*CRC Department of Cancer Genetics, Paterson Institute for Cancer Research;* [2]*CRC Paediatric and Familial Cancer Research Group;* [3]*CRC Psychological Medicine Group;* [4]*CRC Department of Medical Oncology, Christie Hospital, Manchester M20 9BX, UK.*

**Summary** Women with a family history of breast cancer generally self-refer because they have a feeling that their risk is high. However, they have, in general, only a hazy notion of the population risk of breast cancer and their own risk in relation to this. It is probable that they are helped by genetic counselling and, if at substantial risk, by annual mammography. However, the psychological impact of assigning true risk and the value of mammography need to be evaluated. We have assessed risk perception by questionnaire in 517 new referrals to a family history clinic and 200 women returning to the clinic at least 1 year after counselling. Correct assignment of population lifetime risk of breast cancer was 16% in the uninformed precounsel group and 33% in the post-counsel group, likewise personal risk was correct in 11% and 41% respectively. Post-counsel women were significantly more likely to retain information if they were sent a post-clinic letter or if they assessed their personal risk as too high initially.

Demand for information on cancer risks and screening options is growing rapidly as the importance of family history in certain forms of cancer has been demonstrated. Approximately 8% (Solomon, 1990) of colorectal and 4% (Newman *et al.*, 1988) of breast cancer is thought to be caused by dominantly inherited genes. Awareness of breast cancer and its familial nature in a proportion of cases has been heightened by the commencement of the National Breast Screening Programme following the Forrest Report (Working Party on Breast Screening, Forrest report 1986). In response to demand, several specialised regional family history clinics have been set up throughout the UK and clinicians also see individuals on an informal basis. However, there has previously been little information on how women perceive the risk of breast cancer in the general population, or how they feel their risks are altered if they have a family history of the disease. We have recently undertaken a pilot study to assess this (Evans *et al.*, 1993). Since that time we have extended the scope of the original study and assessed the impact of counselling on perceptions of risk and compliance with screening mammography at our breast cancer family history clinic.

### Subjects and methods

### *Subjects*

Referrals to the family history clinic were taken from general practitioners, surgeons and other interested clinicians. Most women who were seen were referred as a result of their own concerns. All women were referred on the basis of at least one affected relative, but the extent of the family history was very variable. Individuals were interviewed by a geneticist (D.G.R.E.) or oncologist (A.H.) and a pedigree constructed (this was partly created prior to the interview from a subject questionnaire). All family cases of breast cancer were recorded including age at onset and uni- or bilaterality. A history of endocrine risk factors including age at menarche, birth of first child and menopause, oral contraceptive use and number of full-term pregnancies was recorded. Dietary, alcohol and smoking habits were also determined. An individual's lifetime risk of developing breast cancer was then estimated based on previous studies (Clauss *et al.*, 1990). The risk given to the individual was expressed as a gambling odds ratio. In most instances a clinic letter was sent to the individual containing a risk assessment and the results of any tests within 2 weeks of the clinic appointment.

### *Procedure*

All new referrals to the clinic from December 1990 were given a questionnaire just prior to their appointment (Table I). The questionnaire was devised for a pilot study previously reported here (Evans *et al.*, 1993). From December 1990 until April 1992 no information about risk was given to the woman prior to filling in the questionnaire. There were 308 women in this 'uninformed' group. After May 1992 each subject was given a one-page letter with their clinic appointment telling them about the clinic. This stated that 1 in 12 women in the general population develop breast cancer in their lifetime. There were 209 women in this 'informed' group. The questionnaire was completed in the waiting room and the clinicians (D.G.R.E., A.H.) were not aware of the results during counselling. The questionnaire was given again to all women when they returned to the clinic for screening at least 1 year after initial risk counselling. After May 1992 all questionnaires also contained a 'don't know' category for questions 1 and 2 (Table I).

The chi-square test was used to compare proportions between groups and McNemar's test to compare changes from pre- to post-counselling in perceived population and personal risks (Siegal & Castellan, 1988).

For the purposes of this study the term 'counselling' is used as it is used in genetic counselling. Broadly, this covers explaining the possible hereditary nature of a condition and the risk to that individual and family of that problem. Options open to the individual are also discussed along with any other relevant agendas.

### Results

The results of the first two questions are expressed in Figure 1 and Tables II–V.

### *Uninformed group*

Only 50/308 individuals (16%) of the uninformed group chose the correct population lifetime risk for breast cancer (Cancer Statistics, 1988). Eight women indicated their own risk as very unlikely despite a strong family history in several

Correspondence: D.G.R. Evans, Department of Medical Genetics, St Mary's Hospital, Manchester M13 0JH, UK.
Received 26 January 1994; and in revised form 20 April 1994.

cases. Twelve women could not ascribe a population risk and 18 women were unable to ascribe a personal risk. Almost a quarter (24.5%) of women could not separate their risk from their choice of population risk despite thinking their own risk increased. A total of 251/308 (81.5%) individuals had discussed their breast cancer risk with relatives, while 151/308 (49%) felt they were at risk of other malignancies. All individuals thought screening would be helpful, except four, and these were not sure.

At their first visit 57/308 (18%) individuals were counselled at an estimated risk of 1 in 3 or greater, 21% at 1 in 4, 8% at 1 in 5, 21% at 1 in 6, 10% at 1 in 8, 6% at 1 in 10 and 15% were thought to be at no excess risk over the general population (less than or equal to population risk of 1 in 12) (Table II); 151/308 (49%) individuals had estimated their risk within 50% of their counselled risk. In general, a subject's estimate of risk increased in line with the extent of her family history. However 17/57 women at 1 in 3 or greater counselled risk underestimated their risk by more than 100% (i.e. <1 in 6). There was no significant difference ($\chi^2 = 2.7$, $P = 0.8$) between the counselled risk groups in perception of increased risk of other cancers.

**Figure 1** Perception of population risk of breast cancer pre- and post-counselling. ■, Precounsel; □, precounsel informed; ▨, post-counsel.

**Table I** Questionnaire to assess risk perception

Q1 What do you feel the risk of developing breast cancer is for **any woman** in the general population?

(a) inevitable; (b) 1 chance in 2; (c) 1 chance in 3; (d) 1 chance in 4; (e) 1 chance in 5; (f) 1 chance in 6; (g) 1 chance in 8; (h) 1 chance in 10; (i) 1 chance in 12; (j) 1 chance in 20; (k) 1 chance in 50; (l) 1 chance in 100; (m) very unlikely

Q2 What do you feel **your** lifetime risk of developing breast cancer is? Choices (a) to (m)

Q3 Have you spoken to other members of your family about breast cancer risk? Y/N

Q4 Do you feel you are at increased risk of developing **other** cancers? Y/N

Q5 Do you think screening will help you? Y/N

*Informed group*

Of the 209 women who had received the population risk of breast cancer as part of information sent prior to their clinic visit, 54 (26%) chose the correct population risk and 130 (62%) were within 50% of the 1 in 12 figure (Table IV); 25/209 chose the correct risk for themselves and 98/209 were within 50%. Sixteen per cent could not separate their choice of personal risk from population risk (Table V), a similar proportion to the 20% in the uninformed group, whereas only 5% had this difficulty post counsel.

*Post-counsel group*

Two hundred women from the uninformed group have completed questionnaires on returning to the clinic for screening at least 1 year later; 78 of these also completed a precounsel questionnaire at their first visit. Only 33 had not received a post-clinic letter or received a letter in which the risks were not specified. These were mainly women seen prior to it being our standard practice to write detailed letters to each woman attending. Thirty-three per cent of the 200 women completing the questionnaire at the follow-up visit chose the correct population risk, and 66.5% were within 50% of it. Personal risk was retained more accurately than population

**Table II** Comparison of perceived and counselled risks and median life-time risk chosen for the population by 308 women at risk of breast cancer as a result of their family history, by counselled risk groups

| *Counselled risk* | *1/3* | *1/4* | *1/5* | *1/6* | *1/8* | *1/10* | *1/12* | *Total* |
|---|---|---|---|---|---|---|---|---|
| Number | 19% (57) | 21% (66) | 8% (24) | 21% (66) | 10% (32) | 6% (32) | 15% (45) | 308 |
| Mean age (years) | 36.7 | 39.9 | 40.7 | 43.3 | 42.8 | 46.8 | 48.2 | 41.4 |
| *Perceived risk* | | | | | | | | |
| Population (median) | 1/12 | 1/12 | 1/10 | 1/20 | 1/12 | 1/20 | 1/12 | |
| Risk for self | | | | | | | | |
| = counselled | 18% (10) | 14% (9) | 12% (3) | 6% (4) | 12% (4) | 22% (4) | 0% | 11% (34) |
| > counselled | 25% (14) | 24% (16) | 37% (9) | 29% (19) | 44% (14) | 39% (7) | 53% (24) | 33% (103) |
| < counselled | 54% (31) | 59% (39) | 37% (9) | 59% (39) | 34% (11) | 39% (7) | 38% (17) | 50% (153) |
| No risk ascribed | 4% (2) | 3% (2) | 12% (3) | 6% (4) | 9% (3) | 0% | 9% (4) | 6% (18) |
| Risk of other cancer increased | 54% (31) | 48% (32) | 37% (9) | 53% (35) | 47% (15) | 44% (8) | 47% (21) | 49% (151) |

**Table III** Comparison of perceived and counselled personal risks chosen for 200 women at increased risk of breast cancer on returning to the clinic a year after counselling, by risk groups

| *Counselled risk* | *1/3* | *1/4* | *1/5* | *1/6* | *1/8* | *1/10* | *1/12* | *Total* |
|---|---|---|---|---|---|---|---|---|
| Number | 50 (25%) | 60 (30%) | 24 (12%) | 48 (24%) | 13 (6%) | 5 (2%) | 0 (0%) | 200 |
| *Perceived risk* | | | | | | | | |
| Risk for self | | | | | | | | |
| = counselled | 44% (22) | 45% (27) | 46% (11) | 33% (16) | 23% (3) | 60% (3) | | 41% (82) |
| > counselled | 12% (6) | 8% (5) | 12% (3) | 29% (14) | 23% (3) | 20% (1) | | 16% (32) |
| < counselled | 40% (20) | 47% (28) | 42% (10) | 35% (17) | 54% (7) | 20% (1) | | 41% (83) |
| No risk ascribed | 4% (2) | 0% (0) | 0% (0) | 2% (1) | 0% (0) | 0% (0) | | 1.5% (3) |
| Risk of other cancer increased | 72% (36) | 43% (26) | 42% (10) | 52% (25) | 54% (7) | 80% (4) | | 54% (108) |

**Table IV** Pre- and post-counsel estimates of population lifetime risk of breast cancer

| | >1 in 8 | 1/8–1/10 | 1/12 | 1/20 | <1 in 20 | Don't know | Total |
|---|---|---|---|---|---|---|---|
| Precounsel | 69 (22%) | 56 (18%) | 50 (16%) | 31 (10%) | 90 (29%) | 121 (4%) | 308 |
| Precounsel[a] | 28 (13%) | 55 (26%) | 54 (26%) | 21 (10%) | 31 (15%) | 20 (9%) | 209 |
| Post-counsel | 11 (5%) | 49 (25%) | 67 (33%) | 17 (8%) | 50 (25%) | 6 (3%) | 200 |

[a]This group was informed of the population risk in preclinic literature.

**Table V** Pre- and post-counsel personal life-time risk estimations

| | Overestimate | | Correct | Underestimate | | | |
|---|---|---|---|---|---|---|---|
| | >150% | 100–150% | 100% | 50–100% | <50% | Don't know | Total |
| Pre-counsel | 71 (23%) | 32 (10%) | 34 (11%) | 75 (24%) | 78 (25%) | 18 (6%) | 308 |
| Pre-counsel[a] | 32 (15%) | 42 (20%) | 25 (12%) | 31 (15%) | 42 (20%) | 37 (18%) | 209 |
| Post-counsel | 10 (5%) | 22 (11%) | 82 (41%) | 26 (13%) | 57 (29%) | 3 (1.5%) | 200 |

[a]This group was informed of the population risk in preclinic literature.

risk, with 82/200 (41%) choosing the counselled figure and 130/200 (65%) within 50% of it.

Many fewer women overestimated their personal (5%) and population risks (5%) by more than 50%, while those underestimating by more than 50% remained largely unchanged (25–29%). However, 36/50 of those counselled at 1 in 3 personal risk though themselves at increased risk of other cancers. Only 11/200 women were unable to separae their own risks from their estimate of population risk; 169/200 women had discussed their risks with their relatives and 108 thought their risks of other cancer were increased. Almost all (199/200) felt that screening with mammography would help them.

Of these 200 women, 33 did not receive a post-clinic letter or their risks were not clearly specified in a letter. These women were mainly those seen prior to it being our standard practice to write detailed letters. They were significantly less likely to ascribe correct risks for the population and themselves [0/33, 5/33 (15%)] than those who did [67/167 (40%), 77/167 (46%), $P<0.00001$ and $P<0.001$ respectively].

*Comparative group*

Of the 78 women who completed both pre and post-counsel questionnaires, six correctly chose the population risk on both, two correctly on the pre but incorrectly on the post, 18 incorrectly on the pre and correctly on the post and 52 incorrectly on both. The post-counsel choices were significantly more accurate than precounsel (McNemar's test, $P = 0.0003$). Significantly more choices from the post-counsel questionnaire were within 50% of the true value (corresponding figures 29, 6, 28, 15, McNemar's test, $P = 0.0002$).

Five of the 78 who completed both questionnaires correctly selected their counselled personal risk on both, one correctly on pre but incorrectly on post, 25 incorrectly on pre but correctly on post and 47 incorrectly on both. The post-counsel choices were significantly more accurate than the precounsel (McNemar's test, $P<0.00001$). Significantly more women selected a personal risk within 50% of the counselled figure on the post-counsel questionnaire (corresponding values 31, 5, 21, 21, McNemar's test, $P = 0.002$).

*Reattendance*

Women were invited to reattend for annual screening on the basis of an increased risk of breast cancer. Attendance for follow-up screening from January to July 1993 was 288/299 (96%) at first visit and 98% overall.

### Discussion

It has been standard practice for many years to counsel individuals on their genetic risks in a statistical way (Pearn, 1973). There are many factors which may influence perception of risk and indeed the interpretation of risks offered at counselling. Only a small proportion of women know the correct lifetime risk of breast cancer for the population and for themselves, despite the fact that they have heightened awareness of risk and self-refer themselves to a family history clinic. We have previously shown that individuals do not necessarily understand the concepts of risk when expressed as a gambling odds (Evans *et al.*, 1993). In spite of recent widespread media coverage our high-risk group did not estimate population risk accurately. For example, 29% of women felt the population lifetime risk of breast cancer was 1 in 50 or lower. There is concern that this group may well be worried by the real figure for population risk and their own risk in relation to this. Even when the correct risk is included in literature sent to women attending the clinic, those correctly ascribing population risk still only increases to 26%. This may be because women either do not read the literature or do not retain the information in the small interval before their clinic attendance.

In keeping with our pilot study (Evans *et al.*, 1993) 11.4% of women thought their own risk of developing breast cancer was 1 in 2 or greater and all of these could be reassured to some extent as the highest risk we would ascribe is less than this figure as they would only have a 50% risk of inheriting a gene fault which does not cause cancer in all carriers. The 25% of women who underestimated their risk by more than 50% could well have been worried by the counselling process as compared with only 23% who could have been reassured because they overestimated their risks by a similar amount.

A year after counselling the population of subjects have a significantly better idea of their own risk and that in the general population. Although this may not be as great as one might expect, their knowledge retention is significantly better if a post-clinic letter explaining risks is sent. There appears to be an important group of women who continue to underestimate risk both in themselves and in the population, but who are still concerned enough to attend for screening (98% of women offered screening reattended). We feel this may be as a denial mechanism, as those who had initially overestimated risks appear to have retained more from the counselling sessions. We are assessing this as part of our ongoing research. Only 5% of women in the postcounsel group overestimated their risk by more than 50% compared with 23% in the precounsel group. Clearly these women who are given reduced risks compared with their estimates appear to be benefitting from the counselling process. Our major concern has been over women who underestimate their risk and may be made anxious by learning their risks are much greater than they thought. The fact that the perception of risk in this group of women remains virtually unchanged at a lower level than actual risk suggests that they are not retaining the information because they would rather not confront it. However, our impression is that this group of women are not made more anxious by the consultation, but do benefit from their participation in screening. We are currently assessing all new referrals psychologically to determine whether this impression is correct. This will also address what women understand by 'lifetime risk' and whether information could be presented to women in a way that would make it more absorbable.

We have also assessed a second cohort of women at their initial visit, but who are yet to return for their annual review. These women were given written information about the population risk of breast cancer prior to their visit. While this enabled them to achieve better scores on population risks, there was little difference in their ability to assess their own risk in relation to this (Table V). We did allow women in the second group the chance of entering 'don't know' (Tables IV and V) for their risks, and this increased the proportion not ascribing a risk from 4% to 9% for population risk and 6% to 18% for personal risk. However, despite over half of the post-counsel women having this option, the figure choosing 'don't know' was small (3% and 1.5%).

Approximately 50% of both the pre- and post-counsel groups felt themselves at increased risk of other cancers. However, 72% of those counselled at 1 in 3 risk (Table III) felt their risks of other cancers were increased on their post-counsel questionnaire. This group contains women whose family history is highly suggestive of a dominant inherited predisposition, and in some of these cases risks of other cancers were clearly delineated and screening arranged. This was the case for women whose families contained cases of ovarian cancer in addition to breast cancer. Despite the fact that other cancer risks are not specifically addressed unless the subject asks or the family pedigree suggests an increased risk, the overall perception of other cancer risk remained the same after counselling as before.

There has been a great deal of recent interest centred around the psychological impact on women of giving estimates of risk and subsequently with the effects of DNA predictive testing (Biesecker *et al.*, 1993). A telephone survey of American women between the ages of 50 and 75 years (Polednak *et al.*, 1991) showed that only 35% of women chose the appropriate lifetime odds for NW America (10%) when given only four choices and 30% of women at risk felt themselves not very or not at all likely to develop the disease. Another recent American telephone study (Lerman *et al.*, 1993) showed that 28% of women with a family history of breast cancer did not feel themselves at risk. Work in the UK on women attending a breast screening centre showed that women underestimated the prevalence of breast cancer (Fallowfield *et al.*, 1990), although this study was not aimed at those specifically with a family history. These studies did not assess risk from degree of family history and women were not asked to give their own lifetime risk as an odds ratio. They are nonetheless useful as they give the background of the risk perception in all women with a family history as well as those without. Our concern has been largely with these growing proportion of women who do not self-refer, but who because of increased awareness of their doctors are sent to family history clinics. We feel that these women are more likely to be worried by hearing their own estimated risks.

We have reported a very high compliance rate with the annual screening we offer. This differs from the American studies (Vogel *et al.*, 1991; Lerman *et al.*, 1993), in which it is somewhat surprising that attendance at screening has been reported to be no better in those with a family history of breast cancer than those with no history, despite the fact that risk is perceived to be higher (Vogel *et al.*, 1991). This may be because of poorer attendance among those with high anxiety, while attendance in those with moderate levels of anxiety is thought to be increased (Kash *et al.*, 1992). However, the differences in our UK population may be because we are seeing a motivated and largely self-referred group of women in whom there are no cost implications. Not surprisingly adherence in the USA is significantly increased with the level of education received, as health monitoring of any kind is better in those who are more informed (Lerman *et al.*, 1993). However, another study (Kash *et al.*, 1992) that enrolled women who were essentially self-referred had a high drop-out rate (20%), and of those remaining on review adherence was significantly worse in those who perceived their risk to be high (Kash *et al.*, 1992). This was thought to represent a feeling of powerlessness in the high perceived risk/high anxiety group. While we try not to be totally directive in offering screening and discuss the shortcomings of mammography in some detail, our adherence rates are extremely high. This may reflect an international difference in character or our ability to reduce anxiety and risk perception in the high perceived risk group. Virtually all women attending our clinic feel that they will be helped by screening, but we are not sure whether this is because of reduction in anxiety or that they feel their risks are reduced. The American study by Kash *et al.* (1992) also highlighted the possible need for counselling in 27% of women at high risk, in view of the level of their psychological distress.

Owing to recent breakthroughs implicating at least two genes in hereditary breast cancer (Hall *et al.*, 1990; Malkin *et al.*, 1990), genetic tests are now being offered to individuals in suitable families to determine whether or not they have inherited a gene predisposing to breast cancer. This will have to be approached carefully and psychological evaluation of the effects of learning about inheritance of genes with a greater than 80% chance of causing breast cancer is needed.

### Conclusions

We are not aware of any studies which have assessed risk perception before and after counselling. We have shown that women retain information at least a year after counselling and that this is enhanced by sending them a post-clinic letter. Women are more likely to retain this risk if it represents 'good news' in terms of their previous perceptions.

Adherence with screening is very high in our high-risk group and is not influenced by the alteration of perceived risks. Counselling does not appear to alter knowledge or screening behaviour in women who initially express a low perceived risk. We feel that women benefit from the opportunity to discuss their risks and to have the opportunity for regular surveillance. We are now assessing in more detail the psychological impacts on women referred to the high-risk clinic.

### References

BIESECKER, B.B., BOENNKE, B., CALZONE, K., MARKEL, D.S., GARBER, J.E., COLLINS, F.S. & WEBER, B.L. (1993). Genetic counselling for families with inherited susceptibility to breast and ovarian cancer. *JAMA*, **269**, 1970–1974.

CANCER STATISTICS (1988). *Registration England and Wales 1984*. HMSO: London.

CLAUSS, E.B., RISCH, N.J. & THOMPSON, W.D. (1990). Age at onset as an indicator of familial risk of breast cancer. *Am. J. Epidemiol.*, **131**, 961–972.

EVANS, D.G.R., BURNELL, L.D., HOPWOOD, P. & HOWELL, A. (1993). Perception of risk in women with a family history of breast cancer. *Br. J. Cancer*, **67**, 612–614.

FALLOWFIELD, L.J., RODWAY, A. & BAUM, M. (1990). What are the psychological factors influencing attendance, non-attendance and re-attendance at a breast screening centre? *J. R. Soc. Med.*, **83**, 547–551.

HALL, J.M., LEE, M.K., NEWMAN, B., MORROW, J.E., ANDERSON, L.A., HUEY, B. & KING, M.-C. (1990). Linkage of familial early onset breast cancer to chromosome 17q21. *Science*, **250**, 1684-1689.

KASH, K.M., HOLLAND, J.C., HALPER, M.S. & MILLER, D.G. (1992). Psychological distress and surveillance behaviors of women with a family history of breast cancer. *J. Natl Cancer Inst.*, **85**, 1074-1080.

LERMAN, C., DALY, M., SANDS, C., BALSHEM, A., LUSTBADER, E., HEGGAN, T., GOLDSTEIN, L., JAMES, J. & ENGSTROM, P. (1993). Mammography adherence and psychological distress among women at risk for breast cancer. *J. Natl Cancer Inst.*, **85**, 1074-1080.

MALKIN, D., LI, F.P., STRONG, L.C., FRAUMENI, J.F., NELSON, C.E., KIM, D.H., KASSEL, J., GRYKA, M.A., BISCHOFF, F.Z., TAINSKY, M.A. & FRIEND, S.H. (1990). Germ line p53 mutations in a familial syndrome of breast cancer, sarcomas and other neoplasms. *Science*, **250**, 1233-1238.

NEWMAN, B., AUSTIN, M.A., LEE, M. & KING, M.-C. (1988). Inheritance of breast cancer: evidence for autosomal dominant transmission in high risk families. *Proc. Natl Acad. Sci. USA*, **85**, 3044-3048.

PEARN, J.H. (1973). Patients' subjective interpretation of risks offered in genetic counselling. *J. Med. Genet.*, **10**, 129-134.

POLEDNAK, A.P., LANE, D.S. & BURG, M.A. (1991). Risk perception, family history and use of breast cancer screening tests. *Cancer. Detect. Prev.*, **15**, 257-263.

SIEGEL, S. & CASTELLAN, Jr, N.J. (1988). *Non-parametric Statistics for the Behavioural Sciences*. McGraw-Hill: Singapore.

SOLOMON, E. (1990). The colorectal cancer genes. *Nature*, **343**, 412-413.

VOGEL, V.G., GRAVES, D.S., VERNON, S.W., LORD, J.A., WINN, R.J. & PETERS, G.N. (1991). Mammographic screening of women with increased risk of breast cancer. *Cancer*, **66**, 1613-1620.

WORKING PARTY ON BREAST CANCER SCREENING (1986). *Report to the Health Ministers of England and Wales, Scotland and Northern Ireland*. HMSO: London.